# Postoperative pain and the gut microbiome

**DOI:** 10.1016/j.ynpai.2021.100070

**Published:** 2021-08-03

**Authors:** David Brenner, George D. Shorten, Siobhain M. O'Mahony

**Affiliations:** a Department of Anesthesia and Intensive Care Medicine, Cork University Hospital and University College Cork, Ireland; b APC Microbiome Ireland, University College Cork, Cork, Ireland; c Department of Anatomy and Neuroscience, University College Cork, Cork, Ireland

**Keywords:** Postoperative pain, Microbiome, Post surgical pain, Microbiome-gut-brain axis, Stress

## Abstract

In excess of 300 million surgical procedures are undertaken worldwide each year. Despite recognition of the prevalence of postoperative pain, and improvements in pain management techniques, poorly controlled postoperative pain remains a major unresolved challenge globally. An estimated 71% and 51% of patients experience moderate to severe pain after surgery in in-patient and outpatient settings, respectively. Inadequately controlled pain after surgery is associated with significant perioperative morbidity including myocardial infarction and pulmonary complications.

As many as 20–56% of patients develop chronic pain after commonly performed procedures such as hernia repair, hysterectomy, and thoracotomy. Traditional analgesics and interventions are often ineffective or partially effective in the treatment of postoperative pain, resulting in a chronic pain condition with related socio-economic impacts and reduced quality of life for the patient. Such chronic pain which occurs after surgery is referred to as Persistent Post-Surgical Pain (PPSP). The complex ecosystem that is the gastrointestinal microbiota (including bacteria, fungi, viruses, phage) plays essential roles in the maintenance of the healthy state of the host. A disruption to the balance of this microbiome has been implicated not only in gastrointestinal disease but also neurological disorders including chronic pain. The influence of the gut microbiome is well documented in the context of visceral pain from the gastrointestinal tract while a greater understanding is emerging of the impact on inflammatory pain and neuropathic pain (both of which can occur during the perioperative period). The gut microbiome is an essential source for driving immune maturation and maintaining appropriate immune response. Given that inflammatory processes have been implicated in postoperative pain, aberrant microbiome profiles may play a role in the development of this type of pain. Furthermore, the microorganisms in our gut produce metabolites, neurotransmitters, and neuromodulators which interact with their receptors to regulate peripheral and central sensitisation associated with chronic pain. Microbiota-derived mediators can also regulate neuroinflammation, which is associated with activation of microglia as well as infiltration by immune cells, known to modulate the development and maintenance of central sensitisation. Moreover, risk factors for developing postoperative pain include anxiety, depression, and increased stress response. These central nervous system-related disorders have been associated with an altered gut microbiome and microbiome targeted intervention studies indicate improvements. Females are more likely to suffer from postoperative pain. As gonadal hormones are associated with a differential microbiome and pre-clinical studies show that male microbiome confers protection from inflammatory pain, it is possible that the composition of the microbiome and its by-products contribute to the increased risk for the development of postoperative pain. Very little evidence exists relating the microbiome to somatic pain. Here we discuss the potential role of the gut microbiome in the aetiology and pathophysiology of postoperative pain in the context of other somatic pain syndromes and what is known about microbe-neuron interactions. Investigations are needed to determine the specific role of the gut microbiome in this type of pain which may help inform the development of preventative interventions as well as management strategies to improve patient outcome.

## Highlights

• Poorly controlled postoperative pain remains a major unresolved challenge globally.
• The gut microbiome impacts on inflammatory pain and neuropathic pain.
• Microbiota metabolites can regulate peripheral and central sensitisation.
• Stress is linked to both postoperative pain and an altered gut microbiome.

## Introduction

The International Association for the Study of Pain defines pain as “an unpleasant sensory and emotional experience associated with actual or potential tissue damage, or described in terms of such damage” (IASP, 1979). This physiological/pathophysiological phenomenon serves as a protective measure against mechanical/chemical/thermal insults. How we react to pain depends on a complex mechanism embedded in a multi-compartmental system first referred to as the “neuromatrix” by Melzack, 1999, Melzack, 2005. The input of the neuromatrix consists of sensory (peripheral nervous system), affective (limbic system, etc.), and cognitive (memories, attention, etc.) elements. While output of this matrix regulates motor regions of the brain (both involuntary and voluntary), stress regulation and pain perception (Melzack, 1999).

The most common triggers of somatic pain are traumatic injuries and surgery. Extensive research has been conducted over decades into the nature and characteristics of surgical pain. Pre-clinical studies have been an important element of this research. Early animal models of incisional pain i.e. plantar incision in rodents representing surgical trauma (Brennan et al., 1996), did not completely represent the complex pathophysiology of surgical pain. While, more recent animal models attempt to more accurately represent the entire trauma of surgery and include further elements associated with the actual surgical procedure, such as including the impact of muscle retractors (Flatters, 2008). Most recently, porcine models of surgical pain have been studied with the obvious benefit of closer phylogenetic proximity, and the ability to examine the effectiveness of topical and localized treatments (Castel et al., 2014). Translational studies in human models of surgical pain also highlight the potential pathophysiology of this type of pain (Fimer et al., 2011, Pogatzki-Zahn et al., 2010). Pogatzki-Zahn and colleagues investigated changes in brain activation (functional MRI) during incisional pain in healthy volunteers showing changes in the secondary somatosensory cortex, frontal cortex, and the limbic system (Pogatzki-Zahn et al., 2010).

Sensitisation (neuronal plasticity) occurs in response to painful stimuli. Under physiological conditions, sensitisation is part of the normal defence mechanisms of the body against repeated noxious stimuli. It also plays an important role in the development of chronic pain. Sensitisation is a complex phenomenon of which detailed discussion is beyond the scope of this article. We refer to the excellent reviews by Woolf and Salter, and Cohen and Mao (Cohen and Mao, 2014, Woolf and Salter, 2000). Sensitization can occur at peripheral (autosensitisation of nociceptive terminals, see above) and central level.

Important mechanisms which result in central sensitisation in the spinal cord include receptive field expansion (heterosynaptic potentiation by low frequency nociceptor inputs) and windup: repeated or intense stimulation results in release of glutamate, aspartate and different neuromodulators (sP, CGRP, etc.) with consequent slow excitatory postsynaptic potentials and NMDA / non-NMDA receptor activation, increased intracellular calcium concentration and prostaglandin formation (Latremoliere and Woolf, 2009). Modulation of pain pathways by phosphorylation of receptors and ion channels increase the responsiveness of neurons (activity dependent enhancement of transmission).

Another feature of neuronal plasticity is the modification of the neuronal phenotype occurring after inflammation or axon damage (Woolf and Salter, 2000). Target derived growth factors and changes in gene expression are responsible for these changes. Central sensitisation can also result from disinhibition: long-lasting depression of descending inhibitory pathways. As sensitisation occurs via multiple signal molecules, inhibition of only one pathway may not be enough to eliminate sensitisation. This could explain why treatment of acute and chronic pain is so challenging in certain patients - pain is not the simple binary response to a noxious stimulus produced by a “wire network” but a complex and plastic process whereby multiple changes determine the net gain of the system (Latremoliere and Woolf, 2009).

## Persistent post-surgical pain

Persistent Post-Surgical Pain (PPSP) can affect as many as 10–50% of patients undergoing breast, cardiothoracic, limb amputation or inguinal hernia surgery (Neil and Macrae, 2009). Severe, debilitating pain can occur in 2–10 percent of these patients (Kehlet et al., 2006). As these procedures are commonly performed, the socio-economic impact is also significant (Gan et al., 2014).

The reason why certain people develop PPSP is not completely understood; very often common analgesics used as part of multimodal analgesic regime (NSAIDS, opiates) are ineffective in its treatment (Breivik, 1998). Postoperative pain is primarily nociceptive and inflammatory in origin with transient peripheral and central sensitisation subsiding in the days or weeks after surgery (Gulur and Nelli, 2019). But when pain persists longer than 60–90 days it transitions into chronic or persistent postsurgical pain (Gulur and Nelli, 2019).

When the surgical approach predisposes (e.g. rib retractors in open thoracic surgery; radical mastectomy or axillary block dissection) or traumatic tissue handling occurs, nerve injury and consequent neuropathic pain can develop (Gerner, 2008). Some of the pathophysiological elements associated with neuropathic pain following nerve injury are: (i) injured and neighbouring peripheral nerves’ spontaneous ectopic activity; (ii) hypersensitivity of dorsal horn neurons caused by microglia cells (Jin et al., 2003), (iii) altered synaptic receptor and transmitter gene expression (Martin et al., 2019), (iv) apoptosis of dorsal horn inhibitory interneurons (Scholz et al., 2005), (v) dysfunction of descending pain modulatory circuits (Ossipov et al., 2014). However, many patients with obvious surgical nerve injury recover without persistent pain indicating that nerve injury is necessary but not sufficient for developing PPSP – genetic susceptibility, psychosocial factors, age and gender may also contribute (Kehlet et al., 2006).

The prevention and treatment of persistent postsurgical pain are beyond the scope of this article. Minimally invasive and “nerve sparing” surgical techniques, preventive analgesia and novel pharmacological approaches (alpha-2-delta ligands (Burke and Shorten, 2010), alpha-2-adrenergic agonists, ketamine, intravenous lidocaine (Grigoras et al., 2012), corticosteroids, mexiletine, venlafaxine, etc.) (Chaparro et al., 2013) have become widely applied to decreasing the risk of pain persistence after surgery.

## Neuropathic pain

Neuropathic pain is the consequence of the injury / dysfunction of the nervous system and it is always maladaptive (Cohen and Mao, 2014). Amongst those people suffering from chronic pain (25–30% in Europe), the prevalence of neuropathic pain is approximately 20%, imposing a significant socio-economic burden on the society (Cruccu and Truini, 2017, Leadley et al., 2012). Symptoms of neuropathic pain differ in character compared to those of nociceptive pain. Neuropathic pain is usually described as electric-like, stabbing or lancinating; commonly associated with sensory deficits (numbness or tingling) and autonomic signs. Hypersensitivity, allodynia and unpredictable exacerbations are common (Cohen and Mao, 2014).

The pathophysiology of neuropathic pain is complex. Peripheral mechanisms include (i) peripheral sensitisation due to inflammation (see above); (ii) spontaneous discharge of injured nerves (increase in Type III sodium channels and α_2_δ calcium channel subunit; decrease in µ opioid receptors) (Black et al., 1999, deGroot et al., 1997, Patel and Dickenson, 2016); and (iii) phenotypic switch. Central sensitization (spinal and supraspinal mechanisms) discussed above is also implicated in neuropathic pain (Woolf, 2011). Sensitization usually occurs after nociceptive stimuli, whether it develops into chronic neuropathic pain depends on a myriad of physiological (age, gender, pain intensity and location, co-morbidities, obesity, genetics, etc) and psychological factors (pain attitude, cultural background, history of abuse, physical activity) (Mills et al., 2019). Neuropathic pain can be the consequence of mono- or polyneuropathy (Marchettini et al., 2006). Mononeuropathic disorders include nerve compressions, nerve injuries, phantom limb pain or complex regional pain syndrome; whereas multiple nerves are affected in diabetic (can cause mono- and multiplex neuropathy too), uremic or toxic neuropathy (Marchettini et al., 2006).

Acute and persistent post-surgical pain (see below) are often accompanied by a neuropathic component (Takenaka et al., 2020). This can be related to any of the above mentioned pathophysiological processes, but nerve injury specific to an anatomical location (e.g. pelvic or thoracic surgery) or multiple surgeries is certainly a contributory element (Park et al., 2018)

## Rebound pain following regional analgesia

The practice of peripheral nerve blockade (PNBs) has increased greatly since the widespread utilisation of the ultrasound in the mid 2000s (Marhofer et al., 2010). However, with the increase in practice of PNBs, studies have indicated certain shortcomings (Abdallah et al., 2015, Harsten et al., 2013, Henningsen et al., 2018). One of these is rebound pain (RP) which may occur in as many as 40% of patients who undergo PNB (Lavand'homme, 2018). Williams and colleagues defined RP as a “quantifiable difference in pain scores when the block is working, versus the increase in acute pain that is encountered during the first few hours after the effects of perineural local anaesthetics resolve” (Williams et al., 2007-Jun;32(3):186–92.). RP has also been described as “a very severe pain when PNB wears off” (Lavand'homme, 2018).

Interestingly, it has been reported that the analgesic effect of a “single shot” nerve block appears to be limited to the first few postoperative hours. After that and up to 12–48 h postoperatively, Galos et al report that patients who have undergone PNB experience more severe pain that those who have undergone GA (Galos et al., 2016). Similarly, health care utilization after discharge from ambulatory wrist fracture surgery was three-fold greater for patients who had undergone PNB compared to those who underwent GA (Galos et al., 2016).

This phenomenon poses questions about the pathophysiology of RP which is likely to be distinct from that of other forms of postoperative pain. Suggested causes include nerve inflammation, the toxic effect of local anaesthetics (Galos et al., 2016); the unopposed nociceptive input when PNB wears off (Harsten et al., 2013); a well-documented hypersensitivity (transient hyoeralgesia) which occurs in this setting also contributes (Sunderland et al., 2016-Feb;41(1):22–7.). The questions of whether (i) RP can be present even in the absence of surgical stimulus and (ii) is it related more to the patient or to the procedure are still unanswered.

Currently, there is no specific treatment for rebound pain. Opiates appear to be ineffective (Dada et al., 2019). Clinicians are required to balancing the risks and benefits of regional techniques and to provide thorough and information to their patients.

## Factors affecting pain following surgery

Over the last three decades, the proportion of all surgery which is performed on an ambulatory basis has increased substantially; in the US more than 60% of surgical procedures are performed on outpatients (Weiser et al., 2016). In developed countries, the proportion of surgical procedures performed on an ambulatory basis continues to increase. The most common complication of day case surgical procedures is postoperative pain, which can result in delayed discharge, increase the needs for medical advice after discharge (phone or unplanned physician visit) and for hospital readmission (Pogatzki-Zahn et al., 2017).

More invasive procedures performed on inpatients i.e., cardiothoracic surgery, major abdominal or orthopaedic surgeries are usually associated with the more severe postoperative pain (Guimaraes-Pereira et al., 2017). Suboptimal analgesia is associated with non-evoked guarding behaviour and consequent immobility (Pogatzki-Zahn et al., 2017). Lack of mobility limits the efficacy of physiotherapy, and it is the main culprit in postoperative pulmonary complications and thromboembolism. Increased morbidity, and prolonged length of hospital stay are significant burden on the patient and the healthcare system (Gan, 2017).

Incisional pain triggers nociceptive, inflammatory and, in certain scenarios, neuropathic mechanisms. Preventive analgesia using a multimodal approach is widely practiced in perioperative medicine. However, even the application of currently available evidence in daily clinical practice can be suboptimal at times. Increased patients’ expectations and overuse of certain pharmacological agents can have deleterious effects such as opioid dependence.

Postoperative pain is one of the most significant “short term fears” of patients awaiting surgery and patients’ pain experience can vary extremely, even if the intensity of surgical stimulus is similar (Joo et al., 2019). Most important determinants of postoperative pain are the type of the surgery, age, anxiety (or other psychological distress), preoperative pain state and medication history (opioids, SSRIs, etc.) (Tighe et al., 2015).

Orthopaedic, thoracic and open abdominal surgeries account for the greatest risk of severe postoperative pain (Gramke et al., 2009). There is a negative correlation between age and postoperative pain / analgesic consumption which can be partially explained by pharmacokinetic and pharmacodynamic changes related to ageing (Gramke et al., 2009). Anxiety, psychological distress (neuroticism, hostility, etc.) and the use of certain coping strategies correlate with postoperative pain and opioid consumption (Caumo et al., 2002). Psychosocial factors including stress, anxiety, depression, catastrophizing, as well as lack of support also contribute to the development of PPSP (Hinrichs-Rocker et al., 2009, Montes et al., 2015). Some studies indicate that implementation of interventions to decrease stress symptoms such as mindfulness may be effective in reducing the risk of PPSP (Wylde et al., 2017) but a substantial amount of work in this area is required to determine the most appropriate stress-reducing interventions. Another important predictor of moderate or severe postoperative pain is the presence of preoperative pain, which may be explained by central sensitisation and differences in pain thresholds. Similarly, the presence of chronic pain and preoperative opioid use, as well as antidepressant medication should alert a clinician to the potential for difficulty in postoperative pain management (Parthipan et al., 2019). Perioperative administration of dexamethasone or pregabalin can be part of the multimodal analgesic approach given their anti-inflammatory or neuro-modulatory properties (pregabalin is an analogue of the inhibitory neurotransmitter GABA and decreases dorsal horn neuron hyperexcitability, hence counteracting sensitisation) (Burke and Shorten, 2010). There are conflicting results in the literature regarding the association of gender and postoperative pain, some reporting more severe pain experience in females (Gramke et al., 2009-Aug;25(6):455–60.). Similarly, greater BMI was only related to increased postoperative pain in isolated studies and other studies have not found any association (Cadish et al., 2017). Recent studies suggest that genetic factors maybe also linked to postoperative pain (Parthipan et al., 2019).

The challenge of identifying those patients who are most at risk of uncontrolled postoperative pain has compelled researchers and clinicians to examine the complex mechanism of postoperative pain and to attempt to develop new treatment strategies. The gut microbiome has been linked to several of the risk factors and mechanisms implicated in developing PPSP (O'Mahony et al., 2017, Rea et al., 2017). Hence, an aberrant microbiome profile which promotes inflammation, stress-related behaviour and pain enhancement may be a novel risk factor for this type of pain. It is tempting to suggest that determining a predictive microbiota signature of PPSP prior to surgery might open up new therapeutic avenues to prevent PPSP.

## The gut microbiome

The gut microbiome is the community of 100 trillion microorganisms including bacteria, archaea, yeast, helminth parasites, and viruses that inhabit our gut (Codagnone et al., 2019) and there is sufficient evidence now that this community of microorganisms contributes to our overall health and well-being (Lynch and Pedersen, 2016). The total number of genes within our microbiome is about 100 times that of our human genome (Tremlett et al., 2017) providing some indication of the power of this ecosystem in host-microbe interactions. Our microbiome is involved in essential functions within the body such as protection from pathogens, host nutrient metabolism, production of vitamins, xenobiotic and drug metabolism, maintenance of structural integrity of the gut mucosal barrier and modulation of the immune system (Valdes et al., 2018). Shaping of the composition of our microorganisms largely begins at birth and several factors influence which microbiota take up residence. These include the mode of delivery, with vaginally and caesarean section born children have divergent microbiota profiles for up to three years of life (Shao et al., 2019). Diet, both during infancy, whether a baby is breast fed or formula fed (Clarke et al., 2014) and adulthood (Dash et al., 2015). Medications such as antibiotics or stress during early life also impact on the microbiota that colonise the gastrointestinal tract (Clarke et al., 2014).

Microbial colonization of the human gastrointestinal tract takes place in parallel with neurodevelopment during the critical developmental window in early life (O'Mahony et al., 2017). Disruption during this early colonization process may lead to the impairment in the hypothalamus–pituitaryadrenal gland (HPA)-axis functioning, maturation of microglia, brain cytokine profile, blood–brain barrier integrity, sensory pathways, alterations in behavior as well as signalling within the entire microbiome-gut-brain axis (Borre et al., 2014, Pronovost and Hsiao, 2019).

Aberrations in the microbiota composition and diminished alpha diversity have been linked to the wide range of somatic disorders including type 2 diabetes and non-alcoholic fatty liver disease (Dabke et al., 2019) as well as and cardiovascular diseases including atherosclerosis, hypertension, and heart failure (Ahmad et al., 2019, van de Wouw et al., 2017. Alpha diversity can be noted as richness, which indicates how many different species could be detected in a microbial ecosystem, and evenness, which indicates if there is equal abundance or do some species dominate others. Evidence also now highlights the role of gut microbiota and gut health in neurological disorders such as depression, anxiety (Winter et al., 2018) as well as traumatic brain injury, Alzheimer’s disease, Parkinson’s disease (Sharon et al., 2016) and chronic pain (Rea et al., 2017).

Many factors including time of day, diet, exercise, smoking, age (Jackson et al., 2016); environment, body mass index (Ottosson et al., 2018), anesthesia (Serbanescu et al., 2019) interactions with other people as well as pets all make a substantial impact on the gut microbiome (David et al., 2014). Yet, despite these constant fluctuations, the overall profile and functional capacity of the gut microbiome of healthy individuals usually returns to its stable baseline (Lozupone et al., 2012).

Although the role of the gut microbiome is well appreciated in the context of visceral pain from the gastrointestinal tract, their involvement in other types of pain such as inflammatory pain, neuropathic pain, migraine, and opioid tolerance is only recently being recognised. Furthermore, the evidence for the involvement of the gut microbiota in pain following surgery is scarce with virtually no investigations into the predictive capacity of the composition and diversity of the microorganisms within our gut in the development of postoperative pain. Yet the mechanisms of pain and microbiome interactions gleaned from studies on other pain types can potentially be translated to the development of PPSP also.

## Microbiome-Gut-Brain axis

Studies in both animals and humans show that the microbiome-gut-brain axis is a bidirectional signalling network linking the brain and the gut microbiota, via various neural, neurotransmitter and molecular signalling mechanisms, such as short-chain fatty acids (SCFA), tryptophan metabolism, cortisol, and immune factors (Cryan et al., 2019). Appropriate functioning of this axis is fundamental to the reciprocal host-microbe relationship.

The neural networks involved in this communication highway include the autonomic nervous system (ANS), the vagus nerve and the enteric nervous system (ENS) within the gut (Cryan et al., 2019). The direct neural communication between gut microbiota and the brain occurs via the vagus nerve where bacteria and their metabolites stimulate afferent neurons of ENS (Forsythe et al., 2014). The vagal signal from the gut can stimulate an anti-inflammatory reflex with afferent signals to the brain initiating an efferent effect where mediators such as acetylcholine are released through interaction with immune cells and reducing/preventing inflammation (Forsythe et al., 2014).

The main neuroendocrine axis, the HPA axis, also plays a vital role in the two-way signalling between the brain and the gut microbiome. Psychological stress causes release of cortisol systemically which impacts on the gut affecting the local environment including altering the microbiome composition. Furthermore, the communication between the gut microbiota and the HPA axis is complex as it is closely linked with other systems, including the gastrointestinal barrier, the immune system, the blood–brain barrier, microbial metabolites, and gut hormones (Farzi et al., 2018). The sensory and autonomic nervous systems are also involved in this communication. The complexity and number of interlinked systems with the HPA axis indicate the importance of the stress system in the microbiome gut brain axis.

The microorganisms within the gut produce a number of different hormones, neurotransmitters as well as SCFAs which can readily enter the systemic blood system to play key roles in communication between the gut microbiota and the nervous system. These are the main metabolites produced by bacterial fermentation of dietary fibre in the gastrointestinal tract and are potent regulators of host energy metabolism and immune functions (Dalile et al., 2019).

As the number of disorders with an altered gut microbiota increases this also implicates aberrant signalling pathways within the microbiome gut brain axis in these diseases which include disorders associated with altered responses to acute and chronic stress, altered nervous system functioning, and exacerbated gut inflammation disorders (Cryan et al., 2019). This highlights the microbiome gut brain axis as potential target for the development of novel therapeutics.

## Microbiota in mediating somatic pain

The products of bacteria including neurotransmitters, metabolites as well as constitutive elements of our gut microbiome are capable of activating nociceptors (Defaye et al., 2020). Pain manifests in different forms with one type being inflammatory pain, such as arthritic pain, which affects a substantial percentage of people world-wide (Boer et al., 2019). A decrease in pain threshold and increase in pain response are seen in inflammatory conditions with mediators such as adenosine 5′-triphosphate (ATP), H^+^_,_ prostaglandin E2 (PGE2), tumour necrosis factor alpha (TNF-α), interleukin 1beta (IL-1β), C–C motif chemokine ligand 2 (CCL2), and chemokine (C-X-C motif) ligand 1 (CXCL1) being released from immune cells (Guo et al., 2019). Hyperalgesia is the enhanced response to a noxious stimulus while allodynia is pain following a non-noxious stimulus, both of which are associated with inflammation. Inflammatory mediators activate or sensitise peripheral nociceptors and may lead to somatic pain hypersensitivity (Cunha et al., 1999). Following this release of mediators, activation downstream signalling pathways occurs which induces phosphorylation in certain receptors and ion channels of first order sensory neurons which can result in peripheral sensitisation (Guo et al., 2019).

The few clinical studies on inflammatory pain and the microbiome indicate that there are associations between the two. A significant association between Streptococcus species (spp.) abundance in stool samples from patients with osteoarthritis, knee pain and inflammation as assessed using magnetic resonance imaging (MRI), independent of obesity was noted (Boer et al., 2019). Differences in beta diversity of the gut microbiota were also noted between groups. *Streptococcus* spp. produce immunogenic bacterial products which they can encapsulate in microvesicles (MV) (Brown et al., 2015)and are capable of initiating macrophage activation through Toll-Like-Receptor pathways. These are the same pathways that are associated with joint inflammation and pain see in osteoarthritis. Beta diversity is the variation of microbial species between samples.

Preclinical studies have used germ free (GF) mice, which are mice born via caesarean section and maintained without any microorganisms, to demonstrate that inflammatory pain requires a full gut microbiome (Amaral et al., 2008). Compared to conventional mice GF displayed reduced pain after induction with carrageen, lipopolysaccharide (LPS), IL-1β, TNF-α as well as CXCL1. An enhanced expression of the anti-inflammatory cytokine IL-10 was associated with this reduced pain state in the GF mice. Colonisation of the GF mice with conventional microbiota saw a return to control levels of inflammation and pain indicating that the microbiome and its metabolites are required for inflammatory pain induction. Neuropathic pain is caused by injury, including nerve damage or chemotherapy drugs, or disease, diabetes for example, which affect the somatosensory nervous system, including both peripheral and central (Costigan et al., 2009). This type of pain is associated with dysesthesia, which is abnormal sensations, or allodynia, which is pain induced by non-painful stimuli (von Hehn et al., 2012) and evidence now highlights the role of the gut microbiome in neuropathic pain (Lin et al., 2020).

Chemotherapy induced peripheral neuropathy is a toxic side effect of some cancer treatment (Staff et al., 2017). Recently the role of the gut microbiome in mediating this pain has been investigated. A complete absence of microbiota as in GF mice as well as a reduction in microbial load produced by antibiotic administration to conventional mice both induced protective effects on oxaliplatin-induced mechanical hyperalgesia (Shen et al., 2017). Infiltration of inflammatory mediators, IL-6 and TNF-α, to the dorsal root ganglia was lower in antibiotic treated mice as was TLR4 in bone marrow, with LPS reversing these anti-inflammatory effects (Shen et al., 2017). This indicates that components of bacterial cell walls can induce the release of pro-inflammatory mediators enhancing oxaliplatin-induced peripheral sensitisation.

Another chemotherapy drug, paclitaxel, administered to mice, was seen to reduce the beneficial bacteria Akkermansia muciniphila which is noted for its protective effects on the gut barrier (Ramakrishna et al., 2019). Furthermore, microglia infiltration into the spinal cord was associated with paclitaxel-induced neuropathic pain sensitivity while a notable absence of infiltrating immune cells was associated with a resistant profile. Moreover, two putatively pain-inhibiting OTUs (Porphyro_2 and Porphyro_16) were negatively associated with microglia in the mouse strain that appeared to be less sensitive to the Paclitaxel-induced pain (Ramakrishna et al., 2019) indicating that gut bacteria play important roles in this type of pain.

Chemotherapy-induced gut toxicity is the phrase that is now associated with the cumulative effects on the gut that are caused by chemotherapy as such as abnormalities in tight junctions, immune dysfunction and changes in the microbiota profile (Bajic et al., 2018). Therefore, it is not surprising that these effects extend to the pain modulatory system also.

Neuropathic pain is also induced by peripheral nerve trauma which can be studied in the pre-clinical setting using the rat model of spared nerve injury (SNI). Using this model, it was noted that an altered gut microbiota composition was associated with anhedonia behaviour in susceptible compared to sham-operated rats or resilient rats (Yang et al., 2019). Anhedonia is a symptom of depression which is often associated with neuropathic pain in patients (McWilliams et al., 2003). It was possible to transfer pain and anhedonia behaviours via microbiota to antibiotic-treated pseudo-germ-free rodents. Furthermore, transplantation of microbiota from the anhedonia resilient rats into significantly improved pain and depression-like phenotypes, including anhedonia (Yang et al., 2019). This study highlights the intertwined relationship of pain, gut microbiome and mental illness suggesting that gut microbiota may be a promising target for improving neuropathic pain management particularly in the context of stress-related co-morbidities.

## Microbiome targeted interventions for somatic pain

Probiotic bacterial species can modulate the composition of the gut microbiota to prevent or improve symptoms of such conditions as inflammatory bowel disease. Such beneficial effects occur as a result of the improved gut epithelial barrier function, cytokine production or specific effects on host biological pathways affected by certain bacterial populations (Quigley, 2019). The development of a probiotic to improve *peri*-operative analgesia would require empiric evidence of an association between particular gut microbiome characteristics (composition, diversity, abundance) with improved clinical outcome or identification of particular host pathways (e.g. serotonin or endocannabinoid) which may be amenable to the influence of microbiome composition. Limited amounts of both categories of evidence exist currently; for the most part it has been acquired using animal models or through secondary outcomes of observational clinical trials. For instance, abundance of specific bacterial species in the gut microbiome are associated with symptom severity in chronic pelvic pain syndrome (Shoskes et al., 2016). Prebiotic and probiotic administration have decreased visceral hypersensitivity in pre-clinical models (Luczynski et al., 2017, Verdu et al., 2006). *Bifidobacterium breve* NCIMB 702,258 administration increases endocannabinoid levels in the liver and epididymal adipose tissue of mice (Patterson et al., 2017) potentially identifying a mechanism of anti-nociceptive action of probiotic bacteria. We suggest that justification exists to explore the potential for development of a pre/probiotic/synbiotic/post-biotic in a targeted fashion for the purpose of meeting the above criteria and thereby improving the quality of perioperative analgesia and comfort.

## Surgery and the gut microbiome

Much of the studies on the gut microbiome and surgery focus on surgery associated with the GI tract. An elegant review by Guyton and Alverdy (2016) provides a comprehensive view of the implications of GI surgery on the microbiome as well as the potential for the gut microbiota profile to lead to post-GI-surgical complications (Guyton and Alverdy, 2017). Despite the paucity of studies of the microbiota and general surgery and vice versa many common interventions that are a necessary part of GI surgery also apply to all types of surgery. These include for example the administration of antibiotics to prevent post-operative infection. Most commonly used are intravenous broad-spectrum antibiotics and oral nonabsorbable antibiotics, these have a substantial impact on the GI microbiome and have lasting effects (Guyton and Alverdy, 2017, Francino, 2015). While antibiotics are a necessary precaution the gut microbiome plays key roles in the host’s resistance to infection, competitively excluding both endogenous and exogenous pathogens (Sassone-Corsi and Raffatellu, 2015) hence modification of the microbiome can also have implications for infection. Moreover, the gut microbiome is a key driver of the local and systemic immune systems and reduction in the host microbial community increases the risk of pathogen infection, and the overgrowth of harmful pathobionts (Kamada et al., 2013).

Medications including anaesthesia also impact on the gut microbiota composition (Jiang et al., 2019, Liufu et al., 2020) with age of induction impacting on the reduction in Lactobacillus species induced by anaesthesia and surgery (Liufu et al., 2020). This reduction in the probiotic species was associated with increased anxiety and altered cognitive behaviours. These behavioural changes as well as the impact on the microbiome were ameliorated by administration of *Lactobacillus salivarius* (Liufu et al., 2020). This highlights the attractive possibility of microbiota manipulation prior to surgery in order to mitigate the potential deleterious impacts on the gut microbiome which is essential to many mechanisms associated with a successful recovery. Duration of surgery as well as different anaesthetic agents can also potentially affect the microbiome differentially.

Furthermore, anxiety and fear of surgery itself leading up to a surgical intervention can modify the microbiome to induce a less resilient composition to deal with the trauma of surgery (Lukovic et al., 2019). Fasting (Paoli et al., 2019), reduced sleep (Krueger and Opp, 2016) and mobility (Mailing et al., 2019) also play roles in gut microbiome modification.

Moreover, while there is very little data or recommendations for pre-operative microbiota targeted strategies to maintain and promote a healthy, resilient gut microbiome further research is warranted given the impact of the numerous pre-operative interventions as well as surgery itself on the gut microbiome which is now being highlighted as playing a role in many systems essential to successful recovery after surgery.

## Potential molecular mechanisms underlying the potentiation of post-surgical pain by gut microbiome

Whilst evidence on the role of the gut microbiome in different types of pain has been reviewed very nicely elsewhere (Defaye et al., 2020, Guo et al., 2019) no data or publications exist for the role of the gut microbiome in the development of post-operative pain. Yet the pathways and mechanisms associated with other types of pain are common to this type of pain also. What is intriguing is the drivers of susceptibility to develop post-operative pain and whether the gut microbiota is among these. The gut microbiome as described above is capable of producing many different mediators and metabolites which when in balance contribute to homeostasis and a healthy functioning host. An imbalance in the gut microbiota composition and hence microbiome gut brain axis signalling may influence the response to surgery, or the interventions associated with surgery and could potentially drive this increased susceptibility in the development of post-operative pain. Both direct and indirect interactions occur between the microbiota and the nervous system allowing commensal bacteria and pathogens to influence sensory neurons.

## Indirect interactions between microbes and the nervous system in pain

Indirect interactions involve activation of the immune response and it is well accepted that upon pathogen detection by the host a rapid immune defensive response is initiated (Defaye et al., 2020, McCusker and Kelley, 2013). Neuro-immune interactions are pivotal in pain whereby the immune system releases a number of key mediators that are capable of nociceptive sensitisation (Chen et al., 2020). Nociceptors differ from other primary sensory neurons as they can become activated in response to noxious or potentially damaging stimuli (Chen et al., 2020). The cell bodies of primary nociceptors are in the dorsal root ganglia as well as the trigeminal ganglia (Lee and Neumeister, 2020) and afferent neurons project to peripheral tissues including viscera, joints, skin and muscle (Lee and Neumeister, 2020). Molecular sensors located at the primary afferent terminals detect physical stimuli such as mechanical injury and noxious temperatures (heat and cold), but are also capable of detecting a plethora of inflammatory mediators (Lee and Neumeister, 2020). Molecular sensors expressed on nociceptors include but are not limited to G-protein coupled receptors (GPCRs), transient receptor potential channels (e.g. TRPA1, TRPV1) sodium channels (e.g. Nav1.7 and Nav1.8), and mechanoreceptors (e.g. Piezo channels) (Ji et al., 2014).

Upon initiation of host defence in response to a microbial infection, recognition by Toll-Like receptors (TLRs) leads to the production of pro-inflammatory mediators such as cytokines (as interleukin-1β (IL-1β), tumor necrosis factor-α (TNF-α) and CCL2) which can activate TRPA1, TRPV1 on nociceptive terminals and lead to neuron depolarisation and action potential along the afferent pain pathways (Pinho-Ribeiro et al., 2017).

The immune response is activated once pathogen associated molecular patterns (PAMPs) are recognised which are localised to the surface of the microorganisms. These cell-wall constituents are relatively conserved in microorganisms including viral and bacterial nucleic acids, lipopolysaccharides (LPS), peptidoglycan (PGN), lipoteichoic acid (LTA), lipoproteins (Medzhitov, 2007)as well as on flagellin (Kumar et al., 2009). The recognition of PGN, a major constituent of the cell walls of both gram-negative and gram-positive bacteria, by TLRs, on macrophages, for example (Wolf and Underhill, 2018)initiates the production of pro-inflammatory cytokines and recruitment of other immune cells such to the site of infection (Defaye et al., 2020). Both LTA, a component of gram-positive bacteria (Wolf and Underhill, 2018) and LPS, a component of gram-negative bacteria are both recognised by TLRs, TLR2 and TLR4 respectively (Lu et al., 2008). LPS induces activation of mitogen-activated protein kinases (MAPKs) as well as transcription factors such as nuclear factor -κB (NF- κB) which leads to an inflammatory cascade pro-inflammatory cytokines and chemokines (Kawai and Akira, 2010). Other contributors to microorganism-induced inflammatory induction include N-formyl peptides via formyl peptide receptors and bacterial flagellin via TLR5 (Defaye et al., 2020). In this way these bacterial components, once recognised by the host immune system, have an indirect impact on pain manifestation but other factors may contribute to the degree of pain. LPS for example has been established as playing a role in inflammatory pain (Hijma et al., 2020). We have previously shown that a genetic predisposition to stress compromised the coordinated CNS response to this peripheral immune activation with a blunted pain response (O'Mahony et al., 2013). Hence, despite the well-established mechanisms of inflammatory induced pain due to microorganisms there are more factors that play a role in this type of pain manifestation.

## Direct interactions between microbes and the nervous system in pain

It has also been shown that bacterial cells produce both constitutive and secreted molecules that are capable of directly activating nociceptive signalling by altering the intrinsic excitability of nociceptive neurons, in the DRG for example (Ochoa-Cortes et al., 2010). Receptors, such as TLR4, which can detect bacterial cell-wall LPS are expressed on DRG neurons (Acosta and Davies, 2008). LPS directly induced sensitisation of TRPV1-mediated capsaicin responses in trigeminal sensory neurons via TLR4 in vitro has also been seen as an example of direct interactions between microbes of sensory neurons (Diogenes et al., 2011). LPS can also activate TRPA1 channels on sensory neurons, without TLR4, leading to neurogenic inflammation. These channels are expressed on extrinsic afferents, enteric neurons and non-neuronal enterochromaffin cells in the gut (Lai et al., 2017) and are more sensitive to LPS than TRPV1 receptors. Another bacterial component that has mechanisms in place to activate sensory neurons are flagellin. These are recognised by TLR5 and NLR family CARD domain-containing protein 4 (NLRC4) with TLR5 being expressed on DRG neurons providing an opportunity for bacterial flagellin to modulate pain signalling (Defaye et al., 2020). Furthermore, it was seen that the capsular polysaccharide A of *Bacteroides fragilis* was sufficient, without the presence of the entire bacteria, to activate sensory neurons (Defaye et al., 2020), shedding light on the significance of an imbalance in the gut microbiota allowing a prevalence in certain species and the development of pain.

While further research is required in order to fully elucidate the direct interactions between gut microbiota constituents and sensory neurons there are several factors that impact the level of influence possible including access the sensory neurons, the expression level of TLRs which are noted to be increased during inflammatory states (De Nardo, 2015), as well as the nature of the bacterial product that translocates beyond the gut and the status of the gut barrier (Lagomarsino et al., 2021).

Gut bacteria products are also capable of influencing the nervous system. Products such as short-chain fatty acids (SCFA), metabolites and neurotransmitters are ligands for nociceptors (Defaye et al., 2020). SCFA are metabolites of fermentation of undigested carbohydrates by microbiota with the colon. Their production is dependent on fibre content of diet and the profile of the bacteria within the colon as some bacteria metabolise specific SCFA and other are involved in the production (Correa-Oliveira et al., 2016). SCFA including butyrate, acetate and propionate can have direct or indirect effects on processes such as cell proliferation, differentiation, and gene expression (Parada Venegas et al., 2019). Butyrate, produced by bacteria such as *Faecalibacterium prausnitzii* and *Ruminococcus bromii* (Louis et al., 2010) is a primary energy source for colonocytes as well as maintaining intestinal homeostasis through anti-inflammatory actions (Correa-Oliveira et al., 2016). The free fatty acid receptor 3 (FFAR3) is activated by SCFA and is expressed in the gastrointestinal tract on enteroendocrine cells as well as post-ganglionic sympathetic and sensory neurons in the autonomic and somatic peripheral nervous system (Nohr et al., 2015) providing opportunities to modulate sensory experience.

SCFA play several important roles within the gut including 5-HT production, pH maintenance to limit pathogens, anti-inflammatory effects including inhibition of NK-κB and maintaining gut epithelial barrier integrity (Wang et al., 2012). Butyrate has been shown to be beneficial in the reduction of visceral pain potentially through 5-HT release as well as deactivation of TRPV1 channels through repeated stimulation (Kannampalli et al., 2011).

Probiotic bacteria, including *Lactobacillus* and *Bifidobacteria* within the gut are capable of producing neurotransmitters such as 5-HT, noradrenaline, gamma-aminobutyric acid (GABA), and histamine among others (Strandwitz, 2018). The role of 5-HT in pain is well documented (Sommer, 2004) and is produced not only by bacteria in the gut but also by enterochromaffin cells. The activation of TRPV4 by 5-HT has been implicated in visceral pain (Cenac et al., 2010) and we have altered 5-HT signalling in a preclinical model of irritable bowel syndrome and (O'Mahony et al., 2010) in patients with this painful syndrome (O'Mahony et al., 2008).

A number of protective mechanism inbuilt into gastrointestinal tract help to modulate the gut microbiome and its influences on the host. For example, NLRC4 activates the inflammasome complex leading to the death of infected cells (Ley and Gewirtz, 2016) which acts as a mechanism to limit the level of flagellated bacteria in the gut. Ly6/Plaur domain containing 8 (Lypd8), expressed on gut epithelial cell, blocks motility of bacteria such as Escherichia coli in the colon, which reduces bacterial access to the epithelium and contributes to microbiome homeostasis (Okumura and Takeda, 2018). Several other bacterial products are capable of indirectly affecting pain processing through the immune system, including microbial anti-inflammatory molecule (MAM) which is associated with *Faecalibacterium prausnitzii* (Quevrain et al., 2016). This anti-inflammatory, commensal is reduced in Crohn’s disease and it inhibits the NF-κB pathway and also modulates the release of IL-10 and IL-12 (Rossi et al., 2015). While other bacteria such as Collinsella which is associated significantly with high levels of alpha-aminoadipic acid and asparagine and the proinflammatory cytokine IL-17A. This bacterium impairs gut permeability and is linked to disease severity in experimental arthritis (Chen et al., 2016).

This highlights that a different gastrointestinal bacterial profile can determine a host’s response to immune challenges and perhaps influence postoperative outcomes including the development of postoperative pain. A more thorough understanding of the mechanisms of direct and indirect interactions between the gut microbiome and sensory neurons is required to uncover novel therapeutic targets to inform therapeutic strategies for postoperative pain.

## Concluding remarks and future directions

Despite clear and substantial inadequacies, there have been no fundamental improvements in the efficacy of postoperative pain management over the past decade. Of the greater than 300 million patients who undergo surgery each year, 30–80% report moderate to severe pain in the first few days postoperatively (Meissner and Zaslansky, 2019). Much of the work in this field has arisen from improved understanding of the relevant pathophysiology and clinical evidence relating to optimisation of existing therapies or has been driven by alterations in the type of surgery being conducted. A multitude of biological changes result from surgery, including inflammation and nerve injury which manifest clinically as acute and persistent pain. Although promising targets such as nerve growth factor and Nav 1.7 have been identified, to date, no new agents with clinical utility have been delivered (McKelvey et al., 2013). Likewise, epigenetics appears to offer strong potential to modify acute and persistent pain after surgery, and the transition from one to the other, although no therapy has yet resulted (Buchheit et al., 2012, Lirk et al., 2015). Measurable improvements in the quality and consistency of perioperative analgesia have been achieved through refined practice of existing techniques such as ultrasound guided regional anaesthesia and continuous wound infusion. Much clinical research has examined the development of improved block techniques or infusion regimens for specific clinical situations with some of these improving analgesic outcome in small but worthwhile increments. Continuous infusion of local anaesthetic to provide long lasting blockade of an anatomical field, plexus, or peripheral nerve have been increasingly studied for benefit after various types of surgery. To date, definitive clinical trials of their efficacy and safety are few, which has limited their widespread uptake in clinical practice (Ilfeld, 2017). In general, the relative benefits of continuous local anaesthetic infusion techniques versus alternatives such as the use of liposomal bupivacaine, and peripheral nerve stimulation remain to be determined. Similarly, many long-established drugs have been “redeployed” for use in perioperative analgesia: these include corticosteroids (De Oliveira et al., 2011), intravenous lidocaine (Grigoras et al., 2012), NMDA antagonists such as ketamine (Laskowski et al., 2011); and the gabapentanoids (Mishriky et al., 2015)and alpha 2 agonists(Blaudszun et al., 2012). Overall, the practice of perioperative analgesia during the past decade is notable for the absence a major advance.

Given the evidence of the influence and substantial potential of gut microbiome to modulate pain through numerous mechanisms it may well be that this complex ecosystem plays essential roles in the development of persistent pain after surgery and may offer the novel therapeutic/mechanism that is neededAs mentioned, the gut microbiome is an essential source for driving immune responses and evidence highlights its role in inflammatory pain and neuropathic pain both of which can occur during the perioperative period and may influence post-operative outcome. Furthermore, the metabolites, neurotransmitters, and neuromodulators produced by the microbiota have the capacity to directly interact with sensory receptors. Further investigations are required to determine if microbiome profiling before surgery can be included as a determinant of the risk of developing postoperative pain.

This is an attractive concept as we know that the gut microbiome is amenable to interventions such as prebiotics, probiotics as well as beneficial dietary changes to include for example high fibre components. We have outlined the capacity of the gut microbiome to induce pain and touched on its role in anti-inflammatory processes as well as maintenance of gut homeostasis and epithelial barrier function. Furthermore, specific bacterial species are also capable of modulating mood and reducing anxiety and stress-related behaviour and markers.

While the notion of pre-operative assessment and modulation of the gut microbiome to improve postoperative outcome is an exciting possibility a substantial amount of further research is required in order to determine the role and mechanisms of the gut microbiome in this type of pain. These future studies will inform the development of microbiome targeted interventions as well as management strategies to improve patient outcome.

## CRediT authorship contribution statement

**David Brenner:** Writing - original draft, Writing - review & editing. **George D. Shorten:** Writing - original draft, Writing - review & editing. **Siobhain M. O'Mahony:** Writing - original draft, Writing - review & editing.

## Declaration of Competing Interest

The authors declare that they have no known competing financial interests or personal relationships that could have appeared to influence the work reported in this paper.

## References

[b0005] Pain terms: a list with definitions and notes on usage. Recommended by the IASP Subcommittee on Taxonomy. Pain. 1979 Jun;6(3):249. 460932

[b0010] Melzack R. From the gate to the neuromatrix. Pain. 1999 Aug;Suppl 6:S121-S6. 10.1016/S0304-3959(99)00145-1 10491980

[b0015] Melzack R, 85–94 (2005). Evolution of the Neuromatrix Theory of Pain. . 2005. 10.1111/j.1533-2500.2005.05203.x 17177754

[b0020] Brennan T.J., Vandermeulen E.P., Gebhart G.F. (1996). Characterization of a rat model of incisional pain. Pain.

[b0025] Flatters S.J. (2008). Characterization of a model of persistent postoperative pain evoked by skin/muscle incision and retraction (SMIR). Pain.

[b0030] Castel D., Willentz E., Doron O., Brenner O., Meilin S. (2014). Characterization of a porcine model of post-operative pain. Eur. J. Pain.

[b0035] Fimer I., Klein T., Magerl W., Treede R.D., Zahn P.K., Pogatzki-Zahn E.M. (2011). Modality-specific somatosensory changes in a human surrogate model of postoperative pain. Anesthesiology.

[b0040] Pogatzki-Zahn E.M., Wagner C., Meinhardt-Renner A., Burgmer M., Beste C., Zahn P.K. (2010). Coding of incisional pain in the brain: a functional magnetic resonance imaging study in human volunteers. Anesthesiology.

[b0045] Cohen S.P., Mao J. (2014). Neuropathic pain: mechanisms and their clinical implications. BMJ.

[b0050] Woolf C.J., Salter M.W. (2000). Neuronal plasticity: increasing the gain in pain. Science.

[b0055] Latremoliere A., Woolf C.J. (2009). Central sensitization: a generator of pain hypersensitivity by central neural plasticity. J. Pain.

[b0060] Neil M.J., Macrae W.A. (2009). Post Surgical Pain- The Transition from Acute to Chronic Pain. Rev Pain..

[b0065] Kehlet H., Jensen T.S., Woolf C.J. (2006). Persistent postsurgical pain: risk factors and prevention. Lancet.

[b0070] Gan T.J., Habib A.S., Miller T.E., White W., Apfelbaum J.L. (2014). Incidence, patient satisfaction, and perceptions of post-surgical pain: results from a US national survey. Curr. Med. Res. Opin..

[b0075] Breivik H. (1998). Postoperative pain management: why is it difficult to show that it improves outcome?. Eur. J. Anaesthesiol..

[b0080] Gulur P., Nelli A. (2019). Persistent postoperative pain: mechanisms and modulators. Curr. Opin. Anaesthesiol..

[b0085] Gerner P. (2008). Postthoracotomy pain management problems. Anesthesiol Clin..

[b0090] Jin S.X., Zhuang Z.Y., Woolf C.J., Ji R.R. (2003). p38 mitogen-activated protein kinase is activated after a spinal nerve ligation in spinal cord microglia and dorsal root ganglion neurons and contributes to the generation of neuropathic pain. J. Neurosci..

[b0095] Martin S.L., Reid A.J., Verkhratsky A., Magnaghi V., Faroni A. (2019). Gene expression changes in dorsal root ganglia following peripheral nerve injury: roles in inflammation, cell death and nociception. Neural Regen Res..

[b0100] Scholz J., Broom D.C., Youn D.H., Mills C.D., Kohno T., Suter M.R. (2005). Blocking caspase activity prevents transsynaptic neuronal apoptosis and the loss of inhibition in lamina II of the dorsal horn after peripheral nerve injury. J. Neurosci..

[b0105] Ossipov M.H., Morimura K., Porreca F. (2014). Descending pain modulation and chronification of pain. Curr. Opin. Support Palliat Care..

[b0110] Burke S.M., Shorten G.D. (2010). Perioperative pregabalin improves pain and functional outcomes 3 months after lumbar discectomy. Anesth. Analg..

[b0115] Grigoras A., Lee P., Sattar F., Shorten G. (2012). Perioperative intravenous lidocaine decreases the incidence of persistent pain after breast surgery. Clin. J. Pain.

[b0120] Chaparro LE, Smith SA, Moore RA, Wiffen PJ, Gilron I. Pharmacotherapy for the prevention of chronic pain after surgery in adults. Cochrane Database Syst Rev. 2013 Jul 24(7):CD008307. 10.1002/14651858.CD008307.pub2 PMC6481826 23881791

[b0125] Cruccu G., Truini A. (2017). Neuropathic pain special interest group of the italian society of n. neuropathic pain: the scope of the problem. Pain Ther..

[b0130] Leadley R.M., Armstrong N., Lee Y.C., Allen A., Kleijnen J. (2012). Chronic diseases in the European Union: the prevalence and health cost implications of chronic pain. J. Pain Palliat Care Pharmacother..

[b0135] Black J.A., Cummins T.R., Plumpton C., Chen Y.H., Hormuzdiar W., Clare J.J. (1999). Upregulation of a silent sodium channel after peripheral, but not central, nerve injury in DRG neurons. J. Neurophysiol..

[b0140] deGroot J.F., Coggeshall R.E., Carlton S.M. (1997). The reorganization of mu opioid receptors in the rat dorsal horn following peripheral axotomy. Neurosci. Lett..

[b0145] Patel R., Dickenson A.H. (2016). Mechanisms of the gabapentinoids and alpha 2 delta-1 calcium channel subunit in neuropathic pain. Pharmacol. Res. Perspect..

[b0150] Woolf C.J. (2011). Central sensitization: implications for the diagnosis and treatment of pain. Pain.

[b0155] Mills S.E.E., Nicolson K.P., Smith B.H. (2019). Chronic pain: a review of its epidemiology and associated factors in population-based studies. Br. J. Anaesth..

[b0160] Marchettini P., Lacerenza M., Mauri E., Marangoni C. (2006). Painful peripheral neuropathies. Curr. Neuropharmacol..

[b0165] Takenaka S., Saeki A., Sukenaga N., Ueki R., Kariya N., Tatara T. (2020). Acute and chronic neuropathic pain profiles after video-assisted thoracic surgery: A prospective study. Medicine (Baltimore).

[b0170] Park J.W., Kim H.S., Yun J.Y., Han I. (2018). Neuropathic pain after sarcoma surgery: prevalence and predisposing factors. Medicine (Baltimore).

[b0175] Marhofer P., Harrop-Griffiths W., Kettner S.C., Kirchmair L. (2010). Fifteen years of ultrasound guidance in regional anaesthesia: part 1. Br. J. Anaesth..

[b0180] Abdallah F.W., Halpern S.H., Aoyama K., Brull R. (2015). Will the real benefits of single-shot interscalene block please stand up? A systematic review and meta-analysis. Anesth. Analg..

[b0185] Harsten A., Kehlet H., Toksvig-Larsen S. (2013). Recovery after total intravenous general anaesthesia or spinal anaesthesia for total knee arthroplasty: a randomized trial. Br. J. Anaesth..

[b0190] Henningsen M.J., Sort R., Moller A.M., Herling S.F. (2018). Peripheral nerve block in ankle fracture surgery: a qualitative study of patients' experiences. Anaesthesia.

[b0195] Lavand'homme P. (2018). Rebound pain after regional anesthesia in the ambulatory patient. Curr. Opin Anaesthesiol..

[b0200] Williams B.A., Bottegal M.T., Kentor M.L., Irrgang J.J., Williams J.P. (2007). Rebound pain scores as a function of femoral nerve block duration after anterior cruciate ligament reconstruction: retrospective analysis of a prospective, randomized clinical trial. Reg. Anesth. Pain Med..

[b0205] Galos D.K., Taormina D.P., Crespo A., Ding D.Y., Sapienza A., Jain S. (2016). Does brachial plexus blockade result in improved pain scores after distal radius fracture fixation? A randomized trial. Clin. Orthop. Relat. Res..

[b0210] Harsten A., Hjartarson H., Werner M.U., Toksvig-Larsen S. (2013). General anaesthesia with multimodal principles versus intrathecal analgesia with conventional principles in total knee arthroplasty: a consecutive, randomized study. J. Clin. Med. Res..

[b0215] Sunderland S., Yarnold C.H., Head S.J., Osborn J.A., Purssell A., Peel J.K. (2016). Regional versus General Anesthesia and the Incidence of Unplanned Health Care Resource Utilization for Postoperative Pain After Wrist Fracture Surgery: Results From a Retrospective Quality Improvement Project. Reg. Anesth. Pain Med..

[b0220] Dada O., Gonzalez Zacarias A., Ongaigui C., Echeverria-Villalobos M., Kushelev M., Bergese S.D. (2019). Does rebound pain after peripheral nerve block for orthopedic surgery impact postoperative analgesia and opioid consumption? A narrative review. Int. J. Environ. Res. Public Health.

[b0225] Weiser T.G., Haynes A.B., Molina G., Lipsitz S.R., Esquivel M.M., Uribe-Leitz T. (2016). Size and distribution of the global volume of surgery in 2012. Bull. World Health Organ..

[b0230] Pogatzki-Zahn E.M., Segelcke D., Schug S.A. (2017). Postoperative pain-from mechanisms to treatment. Pain Rep..

[b0235] Guimaraes-Pereira L., Reis P., Abelha F., Azevedo L.F., Castro-Lopes J.M. (2017). Persistent postoperative pain after cardiac surgery: a systematic review with meta-analysis regarding incidence and pain intensity. Pain.

[b0240] Gan T.J. (2017). Poorly controlled postoperative pain: prevalence, consequences, and prevention. J. Pain Res..

[b0245] Joo J., Moon H.K., Moon Y.E. (2019). Identification of predictors for acute postoperative pain after gynecological laparoscopy (STROBE-compliant article). Medicine (Baltimore)..

[b0255] Tighe P.J., Le-Wendling L.T., Patel A., Zou B., Fillingim R.B. (2015). Clinically derived early postoperative pain trajectories differ by age, sex, and type of surgery. Pain.

[b0260] Parthipan A., Banerjee I., Humphreys K., Asch S.M., Curtin C., Carroll I. (2019). Predicting inadequate postoperative pain management in depressed patients: A machine learning approach. PLoS ONE.

[b0265] Gramke H.F., de Rijke J.M., van Kleef M., Kessels A.G., Peters M.L., Sommer M. (2009). Predictive factors of postoperative pain after day-case surgery. Clin. J. Pain.

[b0270] Caumo W., Schmidt A.P., Schneider C.N., Bergmann J., Iwamoto C.W., Adamatti L.C. (2002). Preoperative predictors of moderate to intense acute postoperative pain in patients undergoing abdominal surgery. Acta Anaesthesiol. Scand..

[b0275] Hinrichs-Rocker A., Schulz K., Jarvinen I., Lefering R., Simanski C., Neugebauer E.A. (2009). Psychosocial predictors and correlates for chronic post-surgical pain (CPSP) – A systematic review. Eur. J. Pain.

[b0280] Montes A., Roca G., Sabate S., Lao J.I., Navarro A., Cantillo J. (2015). Genetic and clinical factors associated with chronic postsurgical pain after hernia repair, hysterectomy, and thoracotomy: a two-year multicenter cohort study. Anesthesiology.

[b0285] Wylde V., Dennis J., Beswick A.D., Bruce J., Eccleston C., Howells N. (2017). Systematic review of management of chronic pain after surgery. Br. J. Surg..

[b0290] Cadish L.A., Hacker M.R., Modest A.M., Rogers K.J., Dessie S., Elkadry E.A. (2017). Association between body mass index and pain following transobturator sling. J. Obstet. Gynaecol..

[b0295] O'Mahony S.M., Clarke G., Dinan T.G., Cryan J.F. (2017). Irritable bowel syndrome and stress-related psychiatric co-morbidities: focus on early life stress. Handb. Exp. Pharmacol..

[b0300] Rea K., O'Mahony S.M., Dinan T.G., Cryan J.F. (2017). The Role of the Gastrointestinal Microbiota in Visceral Pain. Handb. Exp. Pharmacol..

[b0305] Codagnone M.G., Stanton C., O'Mahony S.M., Dinan T.G., Cryan J.F. (2019). Microbiota and Neurodevelopmental Trajectories: Role of Maternal and Early-Life Nutrition. Ann. Nutr. Metab..

[b0310] Lynch S.V., Pedersen O. (2016). The human intestinal microbiome in health and disease. N. Engl. J. Med..

[b0315] Tremlett H., Bauer K.C., Appel-Cresswell S., Finlay B.B., Waubant E. (2017). The gut microbiome in human neurological disease: a review. Ann. Neurol..

[b0320] Valdes A.M., Walter J., Segal E., Spector T.D. (2018). Role of the gut microbiota in nutrition and health. BMJ.

[b0325] Shao Y., Forster S.C., Tsaliki E., Vervier K., Strang A., Simpson N. (2019). Stunted microbiota and opportunistic pathogen colonization in caesarean-section birth. Nature.

[b0330] Clarke G., O'Mahony S.M., Dinan T.G., Cryan J.F. (2014). Priming for health: gut microbiota acquired in early life regulates physiology, brain and behaviour. Acta Paediatr..

[b0335] Dash S., Clarke G., Berk M., Jacka F.N. (2015). The gut microbiome and diet in psychiatry: focus on depression. Curr. Opin. Psychiatry.

[b0340] O'Mahony S.M., Clarke G., Dinan T.G., Cryan J.F. (2017). Early-life adversity and brain development: Is the microbiome a missing piece of the puzzle?. Neuroscience.

[b0345] Borre Y.E., O'Keeffe G.W., Clarke G., Stanton C., Dinan T.G., Cryan J.F. (2014). Microbiota and neurodevelopmental windows: implications for brain disorders. Trends Mol. Med..

[b0350] Pronovost G.N., Hsiao E.Y. (2019). Perinatal Interactions between the Microbiome, Immunity, and Neurodevelopment. Immunity.

[b0355] Dabke K., Hendrick G., Devkota S. (2019). The gut microbiome and metabolic syndrome. J. Clin. Invest..

[b0360] Ahmad A.F., Dwivedi G., O'Gara F., Caparros-Martin J., Ward N.C. (2019). The gut microbiome and cardiovascular disease: current knowledge and clinical potential. Am. J. Physiol. Heart Circ Physiol..

[b0365] van de Wouw M., Schellekens H., Dinan T.G., Cryan J.F. (2017). Microbiota-gut-brain axis: modulator of host metabolism and appetite. J. Nutr..

[b0370] Winter G., Hart R.A., Charlesworth R.P.G., Sharpley C.F. (2018). Gut microbiome and depression: what we know and what we need to know. Rev. Neurosci..

[b0375] Sharon G., Sampson T.R., Geschwind D.H., Mazmanian S.K. (2016). The central nervous system and the gut microbiome. Cell.

[b0380] Jackson M.A., Jeffery I.B., Beaumont M., Bell J.T., Clark A.G., Ley R.E. (2016). Signatures of early frailty in the gut microbiota. Genome Med..

[b0385] Ottosson F., Brunkwall L., Ericson U., Nilsson P.M., Almgren P., Fernandez C. (2018). Connection between bmi-related plasma metabolite profile and gut microbiota. J. Clin. Endocrinol. Metab..

[b0390] Serbanescu M.A., Mathena R.P., Xu J., Santiago-Rodriguez T., Hartsell T.L., Cano R.J. (2019). General anesthesia alters the diversity and composition of the intestinal microbiota in mice. Anesth. Analg..

[b0395] David L.A., Materna A.C., Friedman J., Campos-Baptista M.I., Blackburn M.C., Perrotta A. (2014). Host lifestyle affects human microbiota on daily timescales. Genome Biol..

[b0400] Lozupone C.A., Stombaugh J.I., Gordon J.I., Jansson J.K., Knight R. (2012). Diversity, stability and resilience of the human gut microbiota. Nature.

[b0405] Cryan J.F., O'Riordan K.J., Cowan C.S.M., Sandhu K.V., Bastiaanssen T.F.S., Boehme M. (2019). The microbiota-gut-brain axis. Physiol. Rev..

[b0410] Forsythe P., Bienenstock J., Kunze W.A. (2014). Vagal pathways for microbiome-brain-gut axis communication. Adv. Exp. Med. Biol..

[b0415] Farzi A., Frohlich E.E., Holzer P. (2018). Gut microbiota and the neuroendocrine system. Neurotherapeutics.

[b0420] Dalile B., Van Oudenhove L., Vervliet B., Verbeke K. (2019). The role of short-chain fatty acids in microbiota-gut-brain communication. Nat. Rev. Gastroenterol. Hepatol..

[b0425] Defaye M., Gervason S., Altier C., Berthon J.Y., Ardid D., Filaire E. (2020). Microbiota: a novel regulator of pain. J. Neural Transm. (Vienna).

[b0430] Boer C.G., Radjabzadeh D., Medina-Gomez C., Garmaeva S., Schiphof D., Arp P. (2019). Intestinal microbiome composition and its relation to joint pain and inflammation. Nat. Commun..

[b0435] Guo R., Chen L.H., Xing C., Liu T. (2019). Pain regulation by gut microbiota: molecular mechanisms and therapeutic potential. Br. J. Anaesth..

[b0440] Cunha F.Q., Teixeira M.M., Ferreira S.H. (1999). Pharmacological modulation of secondary mediator systems–cyclic AMP and cyclic GMP–on inflammatory hyperalgesia. Br. J. Pharmacol..

[b0445] Brown L., Wolf J.M., Prados-Rosales R., Casadevall A. (2015). Through the wall: extracellular vesicles in Gram-positive bacteria, mycobacteria and fungi. Nat. Rev. Microbiol..

[b0450] Amaral F.A., Sachs D., Costa V.V., Fagundes C.T., Cisalpino D., Cunha T.M. (2008). Commensal microbiota is fundamental for the development of inflammatory pain. Proc. Natl. Acad. Sci. U.S.A..

[b0455] Costigan M., Scholz J., Woolf C.J. (2009). Neuropathic pain: a maladaptive response of the nervous system to damage. Annu. Rev. Neurosci..

[b0460] von Hehn C.A., Baron R., Woolf C.J. (2012). Deconstructing the neuropathic pain phenotype to reveal neural mechanisms. Neuron.

[b0465] Lin B., Wang Y., Zhang P., Yuan Y., Zhang Y., Chen G. (2020). Gut microbiota regulates neuropathic pain: potential mechanisms and therapeutic strategy. J. Headache Pain.

[b0470] Staff N.P., Grisold A., Grisold W., Windebank A.J. (2017). Chemotherapy-induced peripheral neuropathy: a current review. Ann. Neurol..

[b0475] Shen S., Lim G., You Z., Ding W., Huang P., Ran C. (2017). Gut microbiota is critical for the induction of chemotherapy-induced pain. Nat. Neurosci..

[b0480] Ramakrishna C., Corleto J., Ruegger P.M., Logan G.D., Peacock B.B., Mendonca S. (2019). Dominant role of the gut microbiota in chemotherapy induced neuropathic pain. Sci. Rep..

[b0485] Bajic J.E., Johnston I.N., Howarth G.S., Hutchinson M.R. (2018). From the bottom-up: chemotherapy and gut-brain axis dysregulation. Front. Behav. Neurosci..

[b0490] Yang C., Fang X., Zhan G., Huang N., Li S., Bi J. (2019). Key role of gut microbiota in anhedonia-like phenotype in rodents with neuropathic pain. Transl. Psychiatry.

[b0495] McWilliams L.A., Cox B.J., Enns M.W. (2003). Mood and anxiety disorders associated with chronic pain: an examination in a nationally representative sample. Pain.

[b0500] Quigley E.M.M. (2019). Prebiotics and Probiotics in Digestive Health. Clin Gastroenterol. Hepatol..

[b0505] Shoskes D.A., Wang H., Polackwich A.S., Tucky B., Altemus J., Eng C. (2016). Analysis of gut microbiome reveals significant differences between men with chronic prostatitis/chronic pelvic pain syndrome and controls. J. Urol..

[b0510] Luczynski P., Tramullas M., Viola M., Shanahan F., Clarke G., O'Mahony S. (2017). Microbiota regulates visceral pain in the mouse. Elife.

[b0515] Verdu E.F., Bercik P., Verma-Gandhu M., Huang X.X., Blennerhassett P., Jackson W. (2006). Specific probiotic therapy attenuates antibiotic induced visceral hypersensitivity in mice. Gut.

[b0520] Patterson E., Wall R., Lisai S., Ross R.P., Dinan T.G., Cryan J.F. (2017). Bifidobacterium breve with alpha-linolenic acid alters the composition, distribution and transcription factor activity associated with metabolism and absorption of fat. Sci. Rep..

[b0525] Guyton K., Alverdy J.C. (2017). The gut microbiota and gastrointestinal surgery. Nat. Rev. Gastroenterol. Hepatol..

[b0530] Francino M.P. (2015). Antibiotics and the human gut microbiome: dysbioses and accumulation of resistances. Front. Microbiol..

[b0535] Sassone-Corsi M., Raffatellu M. (2015). No vacancy: how beneficial microbes cooperate with immunity to provide colonization resistance to pathogens. J. Immunol..

[b0540] Kamada N., Seo S.U., Chen G.Y., Nunez G. (2013). Role of the gut microbiota in immunity and inflammatory disease. Nat. Rev. Immunol..

[b0545] Jiang X.L., Gu X.Y., Zhou X.X., Chen X.M., Zhang X., Yang Y.T. (2019). Intestinal dysbacteriosis mediates the reference memory deficit induced by anaesthesia/surgery in aged mice. Brain Behav. Immun..

[b0550] Liufu N., Liu L., Shen S., Jiang Z., Dong Y., Wang Y. (2020). Anesthesia and surgery induce age-dependent changes in behaviors and microbiota. Aging (Albany NY).

[b0555] Lukovic E., Moitra V.K., Freedberg D.E. (2019). The microbiome: implications for perioperative and critical care. Curr. Opin. Anaesthesiol..

[b0560] Paoli A., Tinsley G., Bianco A., Moro T. (2019). The influence of meal frequency and timing on health in humans: the role of fasting. Nutrients.

[b0565] Krueger J.M., Opp M.R. (2016). Sleep and microbes. Int. Rev. Neurobiol..

[b0570] Mailing L.J., Allen J.M., Buford T.W., Fields C.J., Woods J.A. (2019). Exercise and the gut microbiome: a review of the evidence, potential mechanisms, and implications for human health. Exercr. Sport Sci. Rev..

[b0575] McCusker R.H., Kelley K.W. (2013). Immune-neural connections: how the immune system's response to infectious agents influences behavior. J. Exp. Biol..

[b0580] Chen O., Donnelly C.R., Ji R.R. (2020). Regulation of pain by neuro-immune interactions between macrophages and nociceptor sensory neurons. Curr. Opin. Neurobiol..

[b0585] Lee G.I., Neumeister M.W. (2020). Pain: pathways and physiology. Clin. Plast. Surg..

[b0590] Ji R.R., Xu Z.Z., Gao Y.J. (2014). Emerging targets in neuroinflammation-driven chronic pain. Nat. Rev. Drug Discov..

[b0595] Pinho-Ribeiro F.A., Verri W.A., Chiu I.M. (2017). Nociceptor sensory neuron-immune interactions in pain and inflammation. Trends Immunol..

[b0600] Medzhitov R. (2007). Recognition of microorganisms and activation of the immune response. Nature.

[b0605] Kumar H., Kawai T., Akira S. (2009). Toll-like receptors and innate immunity. Biochem. Biophys. Res. Commun..

[b0610] Wolf A.J., Underhill D.M. (2018). Peptidoglycan recognition by the innate immune system. Nat. Rev. Immunol..

[b0615] Lu Y.C., Yeh W.C., Ohashi P.S. (2008). LPS/TLR4 signal transduction pathway. Cytokine.

[b0620] Kawai T., Akira S. (2010). The role of pattern-recognition receptors in innate immunity: update on Toll-like receptors. Nat. Immunol..

[b0625] Hijma H.J., Moss L.M., Gal P., Ziagkos D., de Kam M.L., Moerland M. (2020). Challenging the challenge: a randomized controlled trial evaluating the inflammatory response and pain perception of healthy volunteers after single-dose LPS administration, as a potential model for inflammatory pain in early-phase drug development. Brain Behav. Immun..

[b0630] O'Mahony S.M., Clarke G., McKernan D.P., Bravo J.A., Dinan T.G., Cryan J.F. (2013). Differential visceral nociceptive, behavioural and neurochemical responses to an immune challenge in the stress-sensitive Wistar Kyoto rat strain. Behav. Brain Res..

[b0635] Ochoa-Cortes F., Ramos-Lomas T., Miranda-Morales M., Spreadbury I., Ibeakanma C., Barajas-Lopez C. (2010). Bacterial cell products signal to mouse colonic nociceptive dorsal root ganglia neurons. Am. J. Physiol. Gastrointest. Liver Physiol..

[b0640] Acosta C., Davies A. (2008). Bacterial lipopolysaccharide regulates nociceptin expression in sensory neurons. J. Neurosci. Res..

[b0645] Diogenes A., Ferraz C.C., Akopian A.N., Henry M.A., Hargreaves K.M. (2011). LPS sensitizes TRPV1 via activation of TLR4 in trigeminal sensory neurons. J. Dent. Res..

[b0650] Lai N.Y., Mills K., Chiu I.M. (2017). Sensory neuron regulation of gastrointestinal inflammation and bacterial host defence. J. Intern. Med..

[b0655] De Nardo D. (2015). Toll-like receptors: activation, signalling and transcriptional modulation. Cytokine.

[b0660] Lagomarsino VN, Kostic AD, Chiu IM. Mechanisms of microbial-neuronal interactions in pain and nociception. Neurobiol Pain. 2021 Jan-Jul;9:100056. 10.1016/j.ynpai.2020.100056 PMC7772816 33392418

[b0665] Correa-Oliveira R., Fachi J.L., Vieira A., Sato F.T., Vinolo M.A. (2016). Regulation of immune cell function by short-chain fatty acids. Clin. Transl. Immunol..

[b0670] Parada Venegas D., De la Fuente M.K., Landskron G., Gonzalez M.J., Quera R., Dijkstra G. (2019). Short chain fatty acids (SCFAs)-mediated gut epithelial and immune regulation and its relevance for inflammatory bowel diseases. Front. Immunol..

[b0675] Louis P., Young P., Holtrop G., Flint H.J. (2010). Diversity of human colonic butyrate-producing bacteria revealed by analysis of the butyryl-CoA:acetate CoA-transferase gene. Environ. Microbiol..

[b0680] Nohr M.K., Egerod K.L., Christiansen S.H., Gille A., Offermanns S., Schwartz T.W. (2015). Expression of the short chain fatty acid receptor GPR41/FFAR3 in autonomic and somatic sensory ganglia. Neuroscience.

[b0685] Wang H.B., Wang P.Y., Wang X., Wan Y.L., Liu Y.C. (2012). Butyrate enhances intestinal epithelial barrier function via up-regulation of tight junction protein Claudin-1 transcription. Dig. Dis. Sci..

[b0690] Kannampalli P., Shaker R., Sengupta J.N. (2011). Colonic butyrate- algesic or analgesic?. Neurogastroenterol. Motil..

[b0695] Strandwitz P. (2018). Neurotransmitter modulation by the gut microbiota. Brain Res..

[b0700] Sommer C. (2004). Serotonin in pain and analgesia: actions in the periphery. Mol. Neurobiol..

[b0705] Cenac N., Altier C., Motta J.P., d'Aldebert E., Galeano S., Zamponi G.W. (2010). Potentiation of TRPV4 signalling by histamine and serotonin: an important mechanism for visceral hypersensitivity. Gut.

[b0710] O'Mahony SM, Bulmer DC, Coelho AM, Fitzgerald P, Bongiovanni C, Lee K, et al. 5-HT(2B) receptors modulate visceral hypersensitivity in a stress-sensitive animal model of brain-gut axis dysfunction. Neurogastroenterol Motil. 2010 May;22(5):573-8, e124. 10.1111/j.1365-2982.2009.01432.x 20003079

[b0715] O'Mahony S., Chua A.S., Quigley E.M., Clarke G., Shanahan F., Keeling P.W. (2008). Evidence of an enhanced central 5HT response in irritable bowel syndrome and in the rat maternal separation model. Neurogastroenterol. Motil..

[b0720] Ley R.E., Gewirtz A.T. (2016). Corralling colonic flagellated microbiota. N. Engl. J. Med..

[b0725] Okumura R., Takeda K. (2018). Maintenance of intestinal homeostasis by mucosal barriers. Inflamm. Regen..

[b0730] Quevrain E., Maubert M.A., Michon C., Chain F., Marquant R., Tailhades J. (2016). Identification of an anti-inflammatory protein from Faecalibacterium prausnitzii, a commensal bacterium deficient in Crohn's disease. Gut.

[b0735] Rossi O., Khan M.T., Schwarzer M., Hudcovic T., Srutkova D., Duncan S.H. (2015). Faecalibacterium prausnitzii strain HTF-F and its extracellular polymeric matrix attenuate clinical parameters in DSS-induced colitis. PLoS ONE.

[b0740] Chen J., Wright K., Davis J.M., Jeraldo P., Marietta E.V., Murray J. (2016). An expansion of rare lineage intestinal microbes characterizes rheumatoid arthritis. Genome Med..

[b0745] Meissner W., Zaslansky R. (2019). A survey of postoperative pain treatments and unmet needs. Best Pract Res Clin Anaesthesiol..

[b0750] McKelvey L., Shorten G.D., O'Keeffe G.W. (2013). Nerve growth factor-mediated regulation of pain signalling and proposed new intervention strategies in clinical pain management. J. Neurochem..

[b0755] Buchheit T., Van de Ven T., Shaw A. (2012). Epigenetics and the transition from acute to chronic pain. Pain Med..

[b0760] Lirk P., Fiegl H., Weber N.C., Hollmann M.W. (2015). Epigenetics in the perioperative period. Br. J. Pharmacol..

[b0765] Ilfeld B.M. (2017). Continuous peripheral nerve blocks: an update of the published evidence and comparison with novel. Alternat. Analgesic Modalities. Anesth Analg..

[b0770] De Oliveira G.S., Almeida M.D., Benzon H.T., McCarthy R.J. (2011). Perioperative single dose systemic dexamethasone for postoperative pain: a meta-analysis of randomized controlled trials. Anesthesiology.

[b0775] Laskowski K., Stirling A., McKay W.P., Lim H.J. (2011). A systematic review of intravenous ketamine for postoperative analgesia. Can. J. Anaesth..

[b0780] Mishriky B.M., Waldron N.H., Habib A.S. (2015). Impact of pregabalin on acute and persistent postoperative pain: a systematic review and meta-analysis. Br. J. Anaesth..

[b0785] Blaudszun G., Lysakowski C., Elia N., Tramer M.R. (2012). Effect of perioperative systemic alpha2 agonists on postoperative morphine consumption and pain intensity: systematic review and meta-analysis of randomized controlled trials. Anesthesiology.

